# A Survey on Data Reproducibility in Cancer Research Provides Insights into Our Limited Ability to Translate Findings from the Laboratory to the Clinic

**DOI:** 10.1371/journal.pone.0063221

**Published:** 2013-05-15

**Authors:** Aaron Mobley, Suzanne K. Linder, Russell Braeuer, Lee M. Ellis, Leonard Zwelling

**Affiliations:** 1 Department of Cancer Biology, The University of Texas MD Anderson Cancer Center, Houston, Texas, United States of America; 2 Department of General Internal Medicine, The University of Texas MD Anderson Cancer Center, Houston, Texas, United States of America; 3 Department of Surgical Oncology, The University of Texas MD Anderson Cancer Center, Houston, Texas, United States of America; 4 Department of Experimental Therapeutics, The University of Texas MD Anderson Cancer Center, Houston, Texas, United States of America; National Cancer Center Research Institute, Japan

## Abstract

**Background:**

The pharmaceutical and biotechnology industries depend on findings from academic investigators prior to initiating programs to develop new diagnostic and therapeutic agents to benefit cancer patients. The success of these programs depends on the validity of published findings. This validity, represented by the reproducibility of published findings, has come into question recently as investigators from companies have raised the issue of poor reproducibility of published results from academic laboratories. Furthermore, retraction rates in high impact journals are climbing.

**Methods and Findings:**

To examine a microcosm of the academic experience with data reproducibility, we surveyed the faculty and trainees at MD Anderson Cancer Center using an anonymous computerized questionnaire; we sought to ascertain the frequency and potential causes of non-reproducible data. We found that ∼50% of respondents had experienced at least one episode of the inability to reproduce published data; many who pursued this issue with the original authors were never able to identify the reason for the lack of reproducibility; some were even met with a less than “collegial” interaction.

**Conclusions:**

These results suggest that the problem of data reproducibility is real. Biomedical science needs to establish processes to decrease the problem and adjudicate discrepancies in findings when they are discovered.

## Introduction

The advancement of basic and translational research is dependent upon the validity and reproducibility of findings as published in the scientific literature. The pharmaceutical and biotechnology industries depend on these basic science results from academia as the basis for designing programs of new cancer therapeutics and/or biomarkers. In addition, basic scientists and their trainees use these initial observations as the foundation for their future research projects.

Several recent publications suggested that the seminal findings from academic laboratories could only be reproduced 11–50% of the time [1], [2]. The lack of data reproducibility likely contributes to the difficulty in rapidly developing new drugs and biomarkers that significantly impact the lives of patients with cancer and other diseases.

Recently, the New York Times published an article about the rise of retracted papers in the past few years compared to previous decades [3]. The article states that this larger number may simply be a result of increased availability and thus scrutiny of journal articles due to web access. Alternatively, the article highlighted that the increase in retractions could be due to something much worse; misconduct by investigators struggling to survive as scientists during an era of scarce funding. This latter explanation is supported by another study, which suggested that the most prevalent reason for retraction is misconduct. In their review of all retracted articles indexed in Pubmed (over 2,000 articles) these authors discovered that 67.4% of retracted articles had been retracted due to misconduct [4]. Regardless of the reasons for the irreproducible data, these inaccurate findings may be costing the scientific community, and the patients who count on its work, time, money, and more importantly, a chance to identify effective therapeutics and biomarkers based on sound preclinical work.

This concern about data reproducibility is based on either relatively small samples where the non-reproducibility has been documented (1) or large-scale overviews of the biomedical literature. Direct surveys of a large group of working academic investigators as to their experiences reproducing published results have not been undertaken.

We devised a simple survey to test whether the trends reported in the above referenced manuscripts represent the opinions and experiences of faculty and trainees at a single institution, the University of Texas MD Anderson Cancer Center. There were three main objectives to this study: 1) to estimate the frequency with which the faculty and trainees encounter difficulty in repeating the seminal findings (key points of the paper) from published results in the scientific literature, 2) to determine the response from faculty and trainees when they cannot reproduce the results published in a peer-reviewed manuscript, and 3) to determine what factors underlie the trends identified in our survey.

## Methods

We developed a 20-item anonymous online survey that asked questions related to experiences with reproducing findings from peer-reviewed published manuscripts among MD Anderson faculty and trainees. After general questions to all respondents were answered, trainees (graduate students and postdoctoral fellows) were asked an additional 8 questions concerning their perceived environmental pressure to publish un-validated or suspicious findings from their own laboratory studies. In September 2012, faculty and trainees were emailed an invitation to participate. A reminder email was sent out one week before the online survey was closed. To ensure inclusivity, we invited all University of Texas MD Anderson Cancer Center faculty and trainees to participate. The email invitation provided a link to the study that included a consent statement, description of the study, and the survey items. This study and questionnaire were approved by the University of Texas MD Anderson Cancer Center's Institutional Review Board.

## Results

In our survey of faculty and trainees at the MD Anderson Cancer Center, we found several significant findings that provided insight about data reproducibility, even though the response rate, represented less than 20% of those queried (overall response rate of 14.75% (171/1159) for trainees and 17.16% (263/1533) for faculty). When asked if investigators had ever tried to reproduce a finding from a published paper and not been able to do so, 54.6% (237/434) of all survey respondents said that they had, with 58.5% (154/263) of faculty having experienced the inability to reproduce data, and 48.5% (83/171) of trainees having the same experience (Table 1). Of note, some of the non-repeatable data were published in well-known and respected journals including several high impact journals (impact factor >20).

**Table 1:** Questions to All Respondents.

Questions to All Respondents	% Answered Yes			
	(# Answered Yes/# Who Responded)			
	Total	Senior Faculty	Junior Faculty	Trainee
**Have you ever tried to reproduce a finding from a published paper and not been able to do so?**	**54.6%**	66.2%	48.7%	48.5%
	(237/434)	98/148	(56/115)	(83/171)
**Did you contact the authors who published the findings?**	**78.0%**	79.5%	81.0%	73.1%
	(71/91)	(35/44)	(17/21)	(19/26)
**Were the differences in results ever resolved?**	**33.3%**	37.5%	23.1%	30.8%
	(22/66)	(15/40)	(3/13)	(4/13)
**Did you publish the results that disagreed with those in the literature?**	**33.3%**	48.2%	28.9%	17.6%
	(66/198)	(41/85)	(13/45)	(12/68)
**Did you have difficulty publishing the contradictory results?**	**43.8%**	30.8%	61.5%	66.7%
	(28/64)	(12/39)	(8/13)	(8/12)

Upon finding results from a paper that could not be repeated the question arises; “what to do with that contradictory data?” When survey respondents were asked if they contacted the authors of the research paper, 78% (71/91) said that they had, but the ultimate results of that contact were very mixed (Table 1). 38.5% (25/65) of those who answered this question stated that they received a positive or helpful response from the authors, while 43% (28/65) received a negative or indifferent response; 18.5% (12/65) received no response at all (Table S4). Overall, only 33.3% of respondents were ever able to explain or resolve their discrepant findings. This implies that 66.7% of conflicting results between research groups were not resolved through communication with the authors of the original publication (Table 1). When investigators tried to publish results that contradicted those found in the published literature, 43.8% (28/64) responded that they encountered difficulty, and only 33.3% (66/198) said that they were ever able to have their results published. However, only 17.6% (12/68) of trainees and 28.9% (13/45) of junior faculty published their conflicting data. A larger percentage of senior faculty, 48.2% (41/85), stated they were able to publish their contradictory findings.

The underlying factors that are driving this trend of non-reproducible data may lie within the system itself. When trainees at the institution were asked if they had ever felt pressure to prove the mentors' hypothesis, even when the data that the trainee generated did not support it, 31.4% reported that they had felt pressure (Table 2). Furthermore, 18.6% of trainees said that they had been pressured to publish findings about which they had doubts. Additionally, when asked if they were aware of mentors who required a high impact journal publication before a trainee can complete his or her training in the laboratory of their mentor, 48.9% (68/139) reported that they were aware of this requirement.

**Table 2:** Trainee Only Questions.

Trainee Only Questions	% Yes
	(# Answered Yes/# Responded)
**Were you ever pressured to publish findings of which you had doubt?**	**18.6%**
	(27/145)
**Have you noted pressure from a mentor to prove that his/her hypothesis was correct, even though the data you generated may not support the hypothesis?**	**31.4%**
	(44/140)
**Are you aware of mentors who require a high impact publication before a trainee can leave the lab?**	**48.9%**
	(68/139)

## Discussion

Data reproducibility is fundamental in efforts to advance biomedical research that will ultimately impact the lives of patients. If the initial pre-clinical results are not reliable or reproducible, clinical trials based on these findings are destined to fail. We designed this study in order to determine just how much of a problem data reproducibility is, with a focus on investigators in a single institution. The main objectives of our survey were assessing the frequency, response to, and consequences of lack of data reproducibility. We sent the survey to all faculty and trainees identified in a database at the MDACC. The fact that <20% of personnel responded is likely due to issues related to 1) many queried were not laboratory investigators, and 2) lack of belief that responses would be anonymous, despite being this information included in the email query.

We identified that over half of investigators have had at least one experience of not being able to validate previously reported data. This finding is very alarming as scientific knowledge and advancement are based upon peer-reviewed publications, the cornerstone of access to “presumed” knowledge. If the seminal findings from a research manuscript are not reproducible, the consequences are numerous. Some suspect findings may lead to the development of entire drug development or biomarker programs that are doomed to fail. As stated in our survey, some mentors will continue to pursue their hypotheses based on unreliable data, pressuring trainees to publish their own suspect data, and propagating scientific “myths”. Sadly, when authors were contacted, almost half responded negatively or indifferently. When data are published, the authors must be fully responsible for its integrity, and thus must be prepared to discuss their findings and help investigators troubleshoot experiments when others are unable to reproduce their work. Our survey also provides insight regarding the pressure to publish in order to maintain a current position or to promote ones scientific career. Almost one third of all trainees felt pressure to prove a mentor's hypothesis even when data did not support it. This is an unfortunate dilemma, as not proving a hypothesis could be misinterpreted by the mentor as not knowing how to perform scientific experiments. Furthermore, many of these trainees are visiting scientists from outside the US who rely on their trainee positions to maintain visa status that affect themselves and their families in our country. This statement was observed in our “comments” section of the survey, and it was a finding that provided insight into the far reaching consequences of the pressure to publish. Other comments bring to light an array of concerns from different individuals that could lead to irreproducibility (Table S11).

Our survey addresses the experiences and views (Tables S1, S2, S3, S4, S5, S6, S7, S8, S9, S10, S11) of investigators at one cancer center. Nonetheless, our study strongly supports the previous concerns about data reproducibility and the impact on the field in general, as well as the personal toll on individuals. The inability to validate published data is real and our survey and accompanying comments show that the problem of data reproducibility stems from numerous causes, most being academic expectation of investigators at all levels. In fact, a recent publication demonstrated that retractions are more common in high impact journals compared to less quoted journals [5]. However, the reasons for this observation are likely multifactorial.

A method for correcting the problem was the topic of a recent perspective article where the authors suggested tighter standards on descriptions of methods and the inclusion a universal reporting standard, which should include randomization and blinding of the investigators [6]. Another recent comment on integrity in laboratory research highlights the problem of data falsification and provides insights into how laboratory leaders can avoid misconduct from those who work under their supervision [5]. Additionally, to reduce bias from journal editors or reviewers, a more rigorous review process and higher standards for publication could help to address the problem. While these suggestions may be a start towards identifying the appropriate solution, implementing these changes are challenging. Changes to the “system” will require a concerted effort of journal editors, principal investigators, trainees, institutions, funding agencies, and the public. Although this seems like a daunting task, we have no choice but to address it - the lives of our patients depend on it.

## Supporting Information

Table S1 Additional questions to all respondents.
(DOCX)

Table S2 Additional trainee only questions.
(DOCX)

Table S3 If you had a problem reproducing a finding from a published paper, in which journal was the finding reported?
(DOCX)

Table S4 When you contacted the author how your inquiry was received?
(DOCX)

Table S5 If you did not contact the authors of the original finding, why not?
(DOCX)

Table S6 How were the differences between published data and your data resolved?
(DOCX)

Table S7 If you did not try to publish, why not?
(DOCX)

Table S8 If you have ever felt pressured to publish findings of which you had doubt, then by whom (mentor, lab chief, more advanced post-doc, other)?
(DOCX)

Table S9 Comments about where the pressure to publish questionable findings was coming from.
(DOCX)

Table S10 If you perform an experiment 10 times, what percent of the time must a result be consistent for your lab to deem it reproducible?
(DOCX)

Table S11 Are there any additional comments that you wish us to consider as we analyze the results of this survey?
(DOCX)
