# Supplementary material for: A Survey on Data Reproducibility in Cancer Research Provides Insights into Our Limited Ability to Translate Findings from the Laboratory to the Clinic
Source: PLoS One. 2013 May 15;8(5):e63221. doi: 10.1371/journal.pone.0063221 (PMC3655010; doi:10.1371/journal.pone.0063221)
Supplement: Table S1 — Additional questions to all respondents. (DOCX) [file pone.0063221.s001.docx]

| **TABLE S1** | | | | |
| --- | --- | --- | --- | --- |
| **Questions to All Respondents** | **% Answered Yes** | | | |
|  | **(# Answered Yes / # Who Responded)** | | | |
|  | **Total** | **Senior faculty** | **Junior Faculty** | **Trainee** |
| **Are you senior faculty, junior faculty or trainee (postdoc or graduate student)?** | **100.0%** | 34.1% | 26.5% | 39.4% |
|  | (434) | (148) | (115) | (171) |
| **Did you consider contacting the investigator who published the finding that you could not reproduce?** | **42.5%** | 49.4% | 42.9% | 33.8% |
|  | (90/212) | (44/89) | (21/49) | (25/74) |
| **In any case, were you able to explain the discrepant findings?** | **33.3%** | 35.3% | 34.1% | 30.4% |
|  | (66/198) | (30/85) | (14/44) | (21/69) |
| **Has a pharmaceutical or biotechnology company ever reported to you that a result that you published was not reproducible?** | **2.1%** | 1.5% | 0.9% | 3.3% |
|  | (8/390) | (2/134) | (1/105) | (5/151) |
| **If yes, were the differences in results ever resolved?** | **28.6%** | 50.0% | 0.0% | 20.0% |
|  | (2/7) | (1/2) | (0/0) | (1/5) |
| **Did you try to repeat your own published work?** | **57.1%** | 50.0% | 0.0% | 60.0% |
|  | (4/7) | (1/2) | (0/0) | (3/5) |
| **Were you able to reproduce the results?** | **71.4%** | 100.0% | 0.0% | 60.0% |
|  | (5/7) | (2/2) | (0/0) | (3/5) |
| **(Faculty Only) As a mentor, are you more interested in a candidate for a position in your lab with 4 Cancer Research papers vs one Cancer Cell paper?** | (Faculty only) |  |  |  |
| **4 Cancer Research Papers** | **62.7%** | 64.3% | 60.5% | n/a |
|  | (133/212) | (81/126) | (52/86) |  |
| **1 Cancer Cell Paper** | **37.3%** | 35.7% | 39.5% | n/a |
|  | (79/212) | (45/126) | (34/86) |  |
