# Supplementary material for: A Survey on Data Reproducibility in Cancer Research Provides Insights into Our Limited Ability to Translate Findings from the Laboratory to the Clinic
Source: PLoS One. 2013 May 15;8(5):e63221. doi: 10.1371/journal.pone.0063221 (PMC3655010; doi:10.1371/journal.pone.0063221)
Supplement: Table S2 — Additional trainee only questions. (DOCX) [file pone.0063221.s002.docx]

| **TABLE S2** | |
| --- | --- |
| **Trainee Only Questions** | **% Yes** |
|  | **# Answered Yes /# Responded** |
| **Did your supervisor ever ask you to alter your results or to select the best results which may not be representative of all the results, or suppress some results?** | 15% |
|  | (22/147) |
| **Did you address this with your mentor or supervisor?** | 64% |
|  | (16/25) |
| **Have you ever noted in your laboratory (current or past) selection of the best data for publication, rather than representative data?** | 48.6% |
|  | (68/140) |
