# Supplementary material for: A Survey on Data Reproducibility in Cancer Research Provides Insights into Our Limited Ability to Translate Findings from the Laboratory to the Clinic
Source: PLoS One. 2013 May 15;8(5):e63221. doi: 10.1371/journal.pone.0063221 (PMC3655010; doi:10.1371/journal.pone.0063221)
Supplement: Table S3 — If you had a problem reproducing a finding from a published paper, in which journal was the finding reported? (DOCX) [file pone.0063221.s003.docx]

| **Table S3** | |
| --- | --- |
| **If you had a problem reproducing a finding from a published paper, in which journal was the finding reported?** | |
| Journal | Frequency |
| AACR Journals and others | 1 |
| Acta Oncologica | 1 |
| AJCO | 1 |
| All over from Science/Nature all the way down the impact factor tiers. | 1 |
| American Heart Journal | 1 |
| Bioinformatics | 2 |
| Biological Psychiatry Psychophysiology biological psychology | 1 |
| Biometrics | 1 |
| Blood | 4 |
| Cancer Cell | 2 |
| Cancer Chemotherapy and Pharmacology | 1 |
| Cancer Research | 9 |
| Cell | 6 |
| Cell, Science | 1 |
| Clinical and Experimental Metastasis; JBC; Cancer Cell | 1 |
| Clinical Cancer Research | 1 |
| Clinical investigation | 1 |
| Development | 1 |
| DNA Repair | 1 |
| don't remember | 6 |
| Eucaryotic Cell | 1 |
| Fertiity and Sterility | 1 |
| Gastroenterology | 1 |
| i requested yeast strains in which the tagged protein was improperly made. These strains are all over the literature because many yeast labs use them | 1 |
| Immunology | 1 |
| J Biomol Screen | 1 |
| J Clin Invest. | 2 |
| J Immunol | 1 |
| J of Chromatography B | 1 |
| J. Neuroscience | 1 |
| JBC | 1 |
| JI | 1 |
| Jmc | 1 |
| JNCI | 1 |
| Journal of Biologic Chemistry | 1 |
| Journal of Clinical Endocrinology and Metabolism | 1 |
| Journal of Clinical Oncology | 1 |
| Journal of Health Promotion and Education. | 1 |
| Journal of Immunology | 1 |
| Journal of Nuclear medicine | 1 |
| JVIR | 1 |
| Lancet | 1 |
| Medical Physics | 2 |
| Mol Cell Biol | 3 |
| molecualr cell, science, EMBO, etc | 1 |
| Nature | 10 |
| Nature Cell Biology | 1 |
| Nature Immunology | 4 |
| Nature Medicine | 3 |
| Nature Medicine Lancet Lancet Oncology JNCI JCO Cancer Research Clinical Cancer Research | 1 |
| new england journal of medicine | 2 |
| New England Journal of Medicine Molecular and Cellular Biology Nucleic Acids Research Proceedings of the National Academy of Sciences | 1 |
| no | 1 |
| Plast Reconstr Surg | 1 |
| PNAS | 6 |
| PNAS, Methods in Enzymology, Nature, and other high impact journals | 1 |
| Radiology | 1 |
| Science | 3 |
| Several | 2 |
| Tetrahedron Letters | 1 |
| Third rate journal | 1 |
| this is not journal specific | 1 |
| Total | 240 |
