# Supplementary material for: A Survey on Data Reproducibility in Cancer Research Provides Insights into Our Limited Ability to Translate Findings from the Laboratory to the Clinic
Source: PLoS One. 2013 May 15;8(5):e63221. doi: 10.1371/journal.pone.0063221 (PMC3655010; doi:10.1371/journal.pone.0063221)
Supplement: Table S4 — When you contacted the author how your inquiry was received? (DOCX) [file pone.0063221.s004.docx]

| **Table S4** |
| --- |
| **When you contacted the author how your inquiry was received?** |
| Angrily |
| At first the investigator was agreeable to our concerns, but when asked to do something about the problem, they did not agree that there was a problem. |
| At first they were very nice and worked with us to figure out why we could not reproduce the findings. Over time, when it turned out that we were not the only ones that had problems with the data set, they stopped being responsive and finally are now antagonistic. |
| defensively |
| Did not receive reply to inquiry |
| Fairly well. We talked about why things may have been different in our hands. |
| Generally friendly often didn't have a good answer. |
| He sent a post-doc to MDACC to analyze our data using his methods. I have heard that he is reporting verbally to the community that our data support his findings, which they do not. He has an abstract submitted to an upcoming meeting in which he is claimed further support for his conclusion. I do not know yet whether or not he will claim that our data support it. |
| Hum, "this is what we got and may be a difference in culture conditions, etc....." |
| I do not know, I sent an email |
| I emailed twice. Received no response. |
| I talked to one of authors in person. |
| Ignored |
| Indifference. |
| Initial response usually helpful. I some instances, problem was resolved. More often, inability to reproduce after repeated emails leads to investigators being considerably less helpful; some become hostile. |
| Inquiry was received collegially. Author stands by his results, and we are confident in ours. We are still working to see if there was a difference in methodology that accounts for the different results. |
| Investigator was recalcitrant to consider my concerns. |
| It was well received. We were looking at trying to improve the reliability/consistency of the results (medical device development), and it was not entirely surprising that we were unable to consistently achieve the high water marks presented in the paper. |
| No response |
| no response or helpful technical details |
| not well |
| OK |
| Positively and led to more ideas |
| She reported that the person who did the experiment had since left the lab and she had not been able to reproduce it herself. |
| Surprise that something may be wrong and concern about the reputation of the authors. |
| Suspicious initially, then helpful |
| The author was defensive of their work. I feel that they used suboptimal reagents, without knowing it at the time, which led to the difference. |
| The authors never responded to my inquiry, nor made the reagents available for us to reproduce their findings. |
| The principal investigator fowarded my email to the postdoc who did the experiment. The postdoc gave me few more details than what was described in the article, which didnt help me much |
| They did not respond |
| They just repeated the procedures what they did. |
| They responded promptly and professionally. |
| They were dismissive (this was the Petricoin and Liotta Ovarian Cancer saga back in 2002-2004) |
| Well received, common occurence with variations in analytical detectors/columns |
| Well. We tried to work out the problems together. |
| with offers to help |
| With some not so helpful advice |
| without much concern - reaction was that we were doing something wrong |
