# Supplementary material for: A Survey on Data Reproducibility in Cancer Research Provides Insights into Our Limited Ability to Translate Findings from the Laboratory to the Clinic
Source: PLoS One. 2013 May 15;8(5):e63221. doi: 10.1371/journal.pone.0063221 (PMC3655010; doi:10.1371/journal.pone.0063221)
Supplement: Table S5 — If you did not contact the authors of the original finding, why not? (DOCX) [file pone.0063221.s005.docx]

| **Table S5** |
| --- |
| **If you did not contact the authors of the original finding, why not?** |
| 1. the findings are not important even reproducible 2. biological findings could be context dependent |
| get in trouble |
| I'm only a graduate student. It seemed inappropriate for me to suggest their work was irreproducible. |
| in one case we are still continuing the research |
| It depends if I know the person. If it's an issue with someone I know who is rationale then raising an irreproducible data issue is possible I would not contact someone unless I know them or know how they will likely respond. Many people would interpret such a contact as a shot across the bow. There are enough wars to fight and the NIH does not reward such efforts. |
| Just found out about it last week. Will contact the author next week after double checking our calculations. |
| Reluctant to cause a problem |
