# Supplementary material for: A Survey on Data Reproducibility in Cancer Research Provides Insights into Our Limited Ability to Translate Findings from the Laboratory to the Clinic
Source: PLoS One. 2013 May 15;8(5):e63221. doi: 10.1371/journal.pone.0063221 (PMC3655010; doi:10.1371/journal.pone.0063221)
Supplement: Table S6 — How were the differences between published data and your data resolved? (DOCX) [file pone.0063221.s006.docx]

| **Table S6** |
| --- |
| **How were the differences between published data and your data resolved?** |
| Another group performed studies and found what reported in Cell could be caused by fixation method used, not biological phenotype. |
| I was correct, and demonstrated why the previous report was incorrect. |
| In clinical research, often the problem is the bias in publishing small studies with positive results, which in the end, turn out to be outliers. The true results are general less impressive when a multi center study is done. The second, much less common possibility is fraud, where false data cannot be reproduced. |
| In some cases, clarification from the authors cleared up difficulties and allowed reproducibility. In other cases (more common), after a series of publications and outside investigations, our concerns were determeined to be well founded. The authors had in one case made an error in experimental design that invalidated their findings. In another case, it appears that one of the authors had manipulated the data, and several papers from their group have been retracted. |
| It was my own research that I could not replicate, when I used a larger, and more diverse sample. This was psychosocial research so it may not be relevant to your survey. |
| Letter to the editor with response from the authors. |
| Multiple labs were unable to reproduce the experimental findings and we all published independently. |
| Previous work described the disappearance of a protein after drug treatment. My work showed it simply moved into the nucleus following treatment. Previous work only looked at cytoplasmic protein levels. |
| repeat experiments multiple times ourselves |
| Subsequent publications agreed that we were correct and the original authors were not. So, the scientific system worked correctly - several cases of independent replication of the "real" result were published; the original report was dramatic, but incorrect. |
| The scientific community and regulatory agencies accepted that their results were not sufficient to show efficicacy and new studies were done to try to replicate them, and were not successful. |
| The systems used were different. |
