# Supplementary material for: A Survey on Data Reproducibility in Cancer Research Provides Insights into Our Limited Ability to Translate Findings from the Laboratory to the Clinic
Source: PLoS One. 2013 May 15;8(5):e63221. doi: 10.1371/journal.pone.0063221 (PMC3655010; doi:10.1371/journal.pone.0063221)
Supplement: Table S7 — If you did not try to publish, why not? (DOCX) [file pone.0063221.s007.docx]

| **Table S7** |
| --- |
| **If you did not try to publish, why not?** |
| Am currently finalizing the results and have begun writing the manuscript. |
| anticipated less likely to be accepted |
| B/c we are in the process of obtaining the 'real answer' and will publish findings along with saying that the original report was incorrect. |
| Because I can not use the strain if they are made in error, I do plan on publishing a mutant phenotype that differs from a previous finding in a future paper |
| Because I couldn't get these results with original idea and using other ways instead to approch what I wanted. |
| Because it is very difficult to convince the reviewers that these data are valid, especially if the other paper's PI is well-known in the field |
| Because it was a matter of getting the experiment to work and it never did. Can't publish a failed experiement. |
| Concerns of public battle |
| Couldn't make sense of the data and experiments were not fruitful. |
| Did not have enough data. |
| Difficult to publish negative results. |
| Felt that their results were likely accurate and reproducible and that the error lied with me |
| I could not explain the finding and had no initiative to figure it out. Had other work to do |
| I did not use the exact same materials they used. |
| I do not want to fight with my PI |
| I don't have enough data yet |
| I dont want to waste time to prove that it is really wrong. |
| I ended to think I was the one doing the experiment wrong so preferred not to publish my results |
| I thought I had made a mistake in my methodology |
| I would have been attacked by the authors who had a "bigger" reputation than myself. Not a wise move for a junior investigator |
| It has been many years since the initial publication, the methods, sources, and formulations of the drug are different now than then, so could not be sure the negative findings were not an artifact of the experimental model. |
| It is difficult to publish negative data. |
| It is tricky to publish something that will discredit another researcher. |
| it was a pilot experiment to figure out the experiment conditions, so it could be due to the subtle difference in reagents/ cell cultures,etc |
| It was not a part of my thesis |
| may eventually publish. not a research priority of mine. was working on model building. |
| money, time, etc... |
| More trouble than it would have been worth and preconceived notion of difficulty in publishing conflicting results. |
| negative results aren't publishable |
| negative studies do not get viewed well..this was a single data point, so did not pursue it any further |
| Not part of major project so data was collected but not for publication |
| Not really an avenue to do so. |
| Not worth the aggravation. Dropped the project instead. |
| Not worth the effort to do all the appropriate experiments to rock-solidly prove it wasn't a technical failure, bad reagent etc. Plus the impact of contradicting relatively minor findings is often low or ignored. Rather publish something new. |
| Not worth the effort. |
| Once a concept is established and cited it is very difficult to refute. I have to ask the question: do I want to make refuting other's data a major focus of my group's efforts or move on to other more interesting topics? |
| one negative piece of data does not make a publication. There is no place to publish isolated negative findings that contradict previously published work |
| paper was published in such a high power journal that any discrepancy could be explained by "lab in competence" |
| People build academic careers on random results: if you repeat an experiment until you find the result that you want, sooner or later you will find that result. I have more important things to do than try to educate bad scientists... |
| Problem was their lab error |
| Project is not ready to publish |
| Requires a not of effort to demonstrate the negative results, which could not be used in grant application |
| retrospective clinical study based on clinical data from CT-PET interpretation that may have been equipment or algorithm specific |
| some suttle conditions that are cretic for the experiments may not be includd in our studies |
| submitted to presentation but was a resident and did not have time to write it up |
| the experiment i was redoing wasn't my main objective in my course of study (different origin organ for the cell line used) |
| The finding is in contrary to many of the published findings at the time. Currently, there were some negative/null findings. Therefore, we will begin to look into our data once again and see if there is something there that we did not see in previous attempts. |
| The finding was allowed to die a quiet death. Investigators in the field were aware of the flaw and there were no subsequent manuscripts that built on the incorrect data. It was an honest (if ill-informed) conclusion. |
| The information was not a core of findings. |
| The paper is ready for submission. |
| The project was stopped at the intial stage when i could not get the same data |
| The results were not consistent |
| There should be another option and that is not yet! So, here is the story. We saw the PNAS paper and were quite excited by it. So, we bought the intital reagent to see if the drug works as the authors indicated. Our initial results showed that the results were not reproducible Since, this was a new line of experimentation for us, we concluded that we were doing something worong. So, we repeated the experiment and I had two other trainees (a graduate stduent and a post-doc) repeat the experiment using similar reagents. The results were always the same. Our positive and negative controls worked beautifully, but the experimental did not. So, we examined the problem more and found that the authors have synthesized more potent analogues of the starting compound. We asked the authors for this analogues, but did not get a response. So, we have our translational chemistry lab synthesize these compounds. We next tested the new compounds which corroborated our initial finding. At that point we decided that the only way we can publish our work is we actually find the correct target for these agents. We have identified the correct target and ti is not what the authors had identified it to be. This story will be submitted for publication shortly. |
| This paper described differentiating es cells into b and t cells and didn't work |
| too difficult |
| Too difficult (and also much work) to rebut |
| Too Difficult to rebut. |
| too many other priorities |
| Too much work need to be done to disapprove their data |
| using their data as a positive control. Since it was not working, I just chose another one. |
| Wasn't significant modification in method |
| We are in the process of publishing them. It will likely be a low impact publication, because the field has moved on a bit and may already favor our evidence. |
| We were not interested in publishing because we were working on medical device technology, and didn't want our gains to go into the public domain where competitors could take advantage of our knowledge. |
| will publish soon |
| You cannot publish a negative |
