# Supplementary material for: A Survey on Data Reproducibility in Cancer Research Provides Insights into Our Limited Ability to Translate Findings from the Laboratory to the Clinic
Source: PLoS One. 2013 May 15;8(5):e63221. doi: 10.1371/journal.pone.0063221 (PMC3655010; doi:10.1371/journal.pone.0063221)
Supplement: Table S8 — If you have ever felt pressured to publish findings of which you had doubt, then by whom (mentor, lab chief, more advanced post-doc, other)? (DOCX) [file pone.0063221.s008.docx]

| **Table S8** |
| --- |
| **If you have ever felt pressured to publish findings of which you had doubt, then by whom (mentor, lab chief, more advanced post-doc, other)?** |
| former mentor |
| In the past before coming us |
| mentor |
| Mentor |
| mentor, co-mentor |
| past mentor. quit the lab for this reason. refused to compromise my integrity. |
| Previous PI from another University and lab. |
