# Supplementary material for: A Survey on Data Reproducibility in Cancer Research Provides Insights into Our Limited Ability to Translate Findings from the Laboratory to the Clinic
Source: PLoS One. 2013 May 15;8(5):e63221. doi: 10.1371/journal.pone.0063221 (PMC3655010; doi:10.1371/journal.pone.0063221)
Supplement: Table S9 — Comments about where the pressure to publish questionable findings was coming from. (DOCX) [file pone.0063221.s009.docx]

| **Table S9** |
| --- |
| **Comments about where the pressure to publish questionable findings was coming from** |
| partly, I presented ALL the data collected, especially the one that gave me some doubts in order to address the issue. |
| PI did not care. PI understands that his/her faculty status and current funding level within the department will continue to ensure that his/her position is safe, regardless of any issues that arise in this regard. |
| PI didn't think my concerns were valid and urged me to publish the results in spite of my misgivings. |
| they are the same person. A person above would likely to agree with them. We are the weak point here. |
| toxic relationship- needed the paper to leave |
