# Supplementary material for: A Survey on Data Reproducibility in Cancer Research Provides Insights into Our Limited Ability to Translate Findings from the Laboratory to the Clinic
Source: PLoS One. 2013 May 15;8(5):e63221. doi: 10.1371/journal.pone.0063221 (PMC3655010; doi:10.1371/journal.pone.0063221)
Supplement: Table S10 — If you perform an experiment 10 times, what percent of the time must a result be consistent for your lab to deem it reproducible? (DOCX) [file pone.0063221.s010.docx]

| **Table S10** |
| --- |
| **If you perform an experiment 10 times, what percent of the time must a result be consistent for your lab to deem it reproducible?** |
| (1) experiments in my field are far too expensive to repeat 10 times. (2) this is difficult to answer without more clearly defining what you mean by "consistent". (3) I expect results to be within standard error unless there is a clear explanation for any observed deviation, such as contamination, or other mistakes |
| >60% |
| >70% |
| >8. |
| >80% |
| >80%, you have to consider techinical problem |
| >95% |
| 1 |
| 10 |
| 10 times |
| 100% |
| 100% |
| 100%, and need an explanation for any discrepancies. |
| 3 |
| 4 |
| 41097 |
| 41129 |
| 41130 |
| 41161 |
| 5 |
| 50-60% |
| 6 |
| 60 |
| 7 |
| 7-9 times |
| 70 |
| 70% |
| 75% |
| 8 |
| 8 out of 10 |
| 8 out of 10, providing the 2 failures can be explained |
| 8 times |
| 8 to 10 times |
| 8 to 9 times |
| 80 |
| 80% |
| 80 to 90% |
| 80-90% |
| 80% |
| 80% and above |
| 9 |
| 9 times |
| 9.5 |
| 90 |
| 90-100% |
| 90% |
| 90%- 95% |
| A bit difficult, since most investigators unlikely to perform and experiment 10 times, but I'd say about 7+ |
| a statistically significant number of times, considering all the experiments which were done technically correctly. |
| About 7 out of the 10 times. |
| above 70% |
| all |
| All conditions being equal- 100% |
| All data is within a standard error of mean and is reported as that. The data will never look the same the 10 times that it is replicated. |
| All times unless there is a specific reason (i.e., expired reagent, etc). |
| at least 60% |
| At least 8 |
| at least 8 times |
| depend on what kind of experiment. |
| depends if you have mastered the technique |
| Depends strongly on the type of experiment, but I would say generally 8+. |
| Don't have a lab. |
| Dont't know |
| Early negative data in the series of 10 may only be excluded if adjustments are being made to the experimental model, method, or evolving investigator skill. Otherwise, data from all 10 experiments should be reported. |
| Eight |
| First of all, trying to repeat an experiment 10 times is a wast of time and resource. I trust my data if I can reproduce it three times. |
| For measurements of radiation dose distributions, we expect agreement within 5% or 3 mm 95% of the time. |
| generally 8/10 -- depends on specific assay variability |
| greater than 9 times |
| I do not work in a lab. |
| I don't run a lab, I am a biostatistician, so in terms of data analysis, I would expect the same result each time :) |
| I don't work in a lab. |
| Ideally we would like an explanation for who the results are not consistent and search very hard to find that reason. It would also depend upon how discordant the results are. If there are 1-2 out of 10 that don't match (and we think we know why) then we could throw those out. Otherwise we show all the data with standard deviations. |
| If the appropriate controls work, I would want the experiment to show the same trend 100% of the time. I would accept that the results of each individual experiment would not reach statistical significance 100% of the time but would want the results to be statistically significant 70% of the time (as long as the other 3 experiments clearly show the same trend). |
| If we have replicate experiments that are dissimilar, we first check adhedance to the SOP, and we do it a twice more. If this does not give 3/4 consistent results, we modify procedures and start again. Three consistent results are required for consideration for publication. |
| in case of p53 responsive assay, 100% |
| it depends on what the controls show |
| It should always be consistent - well designed experiments include controls to determine whether there are technical problems with a particular replicate |
| lab: more than 3 time myself: more than 7 time |
| more than 6 |
| My PI's view may be different from mine but I require at least 7 out of 10 times to feel comfortable. |
| n/a |
| N/A |
| N/A (don't run an experimental lab). |
| N/A I do clinical research |
| na |
| No idea |
| Not a reasonable question |
| Not applicable to my research |
| Not applicable to my research interests |
| Not applicable to my work. |
| not in a lab - n/a |
| Not sure we have ever repeated an experiment ten times, but 8 out of 10 would be a rough number. There is consistent (statistically significant), then varying degrees of inconsistency (trending but nonsig p-value, overlapping/no trend, and then significant but in the wrong direction), and the decision to call consistency must also take into account the degree of this inconsistent data. |
| not sure, maybe 3 |
| The result would need to repeat at least 80% of the time. |
| There are often extenuating circumstances. If we can do it the same way and get the same outcome 3 times in a row....that is good |
| two in three times. |
| We try to give the reader a sense of the consistency/ inconsistency, and possible explanations for what might be a correct/ meaningful result but is difficult to reproduce 4 out of 5 times (etc.) |
| we usually don't repeat experiments 10 times |
| You report what you find, regardless of whether it worked 4 out of 10 times, or 9.5-10 out of 10 times. |
| You would like to be able to reproduce it everytime one perfoms the experiment. |
