# Supplementary material for: A Survey on Data Reproducibility in Cancer Research Provides Insights into Our Limited Ability to Translate Findings from the Laboratory to the Clinic
Source: PLoS One. 2013 May 15;8(5):e63221. doi: 10.1371/journal.pone.0063221 (PMC3655010; doi:10.1371/journal.pone.0063221)
Supplement: Table S11 — Are there any additional comments that you wish us to consider as we analyze the results of this survey? (DOCX) [file pone.0063221.s011.docx]

| **Table S11** |
| --- |
| **Are there any additional comments that you wish us to consider as we analyze the results of this survey?** |
| Although I am NTRA, I am a full professor, thus I am quite senior |
| Although I answered Cancer Cell, the candidate would have to convince me of independence and ethics. In this regard, 4 lower tier papers would be preferred if the candidate were smart and performed the work themselves. Separately, the current funding climate, intense focus on productivity (papers, grants), lack of focus on pursuing truth, and not always expert opinions on study sections contribute to a fair amount of weak science - work that is pushed out too quickly and from a "we will prove this hypothesis" perspective, versus "we will test this hypothesis" angle. There is too much focus on fame, money, etc and not enough on academic integrity, at this institution and unfortunately this seems to extend to a majority of US scientific research. |
| authorship and contribution in big papers |
| Communication between PI and postdoc/student in terms of data presentation is key. |
| crumbling of integrity and value - national trend - bean counters judging science by journal names - institutional failure on dealing with alleged fraud - we still have a guy publishing bunch of fake papers around - nearly a year after being exposed publicly. |
| Everything here in US is screwed up. There is nothing to do other than move out. Here is the economy of the market. Who publishes more deserve respect, while others who are honest and cast doubt about their own results (or third party results) as condenmed. There is no way out. It is either join the "bright team" or be labeled as incompetent. |
| Find out how many negative findings/contradictory findings are published. |
| For previous question (#11), it would depend on actual role in the projects- neither or both candidates may be stellar. |
| Fortunately, my current mentor never pushed me to get good data what we need for high impact publication. However, my previous mentor and also our current neighbor lab PI push too much to produce best data all the time. And sometimes it make trainee consider manipulates data only to escape from stress. Especially, many international trainees (postdoc) also have VISA issue. Thus, PI starts push them with visa issue trainees feel a lot of stress and eventually it make them can do whatever PI WANT. |
| From my experience, no one will help you if you stand up for what is right. This becomes a personal issue and not a political talking point. The system is unfortunately broken and no survey can fix the visceral problems we currently face in this forum. |
| I believe the biggest influence of whethe "best" data vs "representative" data is published is due to "lab culture", which is mostly influenced by the PI mentality. Where the leader goes so do the followers. |
| I believe the survey findings may be skewed because the most unscrupulous researchers may not complete the survey (out of fear of being tracked down) or may be inaccurate in some or all of their reporting. |
| I don't run a lab, so perhaps this survey should start by excluding those who don't run a lab. |
| I had to answer the previous question to move on to this page. I'm not actually familiar with the difference between Cancer Research and Cancer Cell. In Biostat/Bioinformatics, the relevant journals for judging a candidate's qualifications are journals in quantitative methodology, not the publications representing a supporting role as analyst. |
| I only answered the last question re. 4 Can Res papers vs. 1 Cancer Cell paper because the survey monkey forced an answer. This is a problem with the survey. Please strike my answer, since I don't agree with either response. It would be 100% context dependent. I could be more impressed in one vs. the other depending on many additional factors. |
| I think that almost every important publication contains at least one minor misinterpretation, because fields are developing at the time. So the thing to concentrate on is whether the major conclusions of the paper are correct or not. |
| I ultimately decided that I did not trust my own results. |
| I'm not qualified to answer #14, as we publish in radiation physics and radiation oncology journals. |
| IMHO, cause for increased retractions is more stringent fact-checking and easier whistle-blowing, including web sites specialized in this (higher detection rate) and increased pressures on lab staff and PIs to publish (to get grants and positions/promotions/tenure). |
| In my experience, IHC data is the most easily skewed to show data the PI wants. Representative samples are almost never shown. |
| in our case the experiment we could not reproduce was a "control" for our experiment. We got the experiment to work, but the "positive control" we set up based on the literature did not work. However, because our experiment confirmed the general phenomena, we did not pursue it further. |
| It is about time someone started this study and I commend your efforts on this! |
| It is important that a clear distinction be made between: 1) deliberate falsification of data and/or poor scientific standards versus 2) honest error |
| Just a comment that whenever we have tried to publish repeated results from other labs, it has been really hard since most journals think its not novel....even if the paper repeats only a part of the results and goes up further from there. |
| Keep up the good work! |
| lack of specific reagents and technologies make many reports unique, and unable to tested for reproducibility until much later. |
| many of the response options are not applicable to non-tenure track clinical faculty who conduct research |
| Most scientists are honorable -a few are not. Are we surprised? |
| My sense is that it is getting harder to "fudge" data, not easier. I think there was more fraud in the past, but less ability to detect it. The increase in retractions may be a result of data being more readily available for re-analysis (in electronic forms). |
| no |
| No |
| NO |
| No. |
| None. |
| Papers published in "high impact" journals often include many types of experiments that together tell a story. However, each experiment is "optimized" and results that support the manuscript are included ("inconsistent results" are discarded). This makes i dufficult to reproduce by independent investigators, even if the protocol is followed exactly as published. |
| Pressure is not from mentors alone, but from the job market and funding dynamics. Pay trainees a livable wage and they will be under much less pressure to publish low quality data. |
| punish those who have power to cover up there misconducts in research. |
| Question 14 is garbage, should be omitted. I am interested in people who publish good work and who are honest and demonstrate integrity at all times. In other words, people not like Dr. Ellis. The review process for journals is flawed and sometimes great work is only accepted in lower impact factor journals and trash is published in higher impact factor journals. |
| Re question 13...I don't really prefer candidates based on their Cell/Cancer Research publication, provided that the post-doc is honest, thoughtful and qualified. Papers are often published in upper tier journals based on the reputation of the PI, not the student. |
| Reproducibility is a crucial issues, and we need to protect the integrity of science. However, we need to be careful not to create a misimpression that all of science or all scientists do non-reproducible research as this can damage the reputation and funding of science. We need to find ways to police ourselves and each other to ensure only the highest quality science sees the light of day, especially in top journals. This means that when someone gets "pie in the sky" and dramatic results, we need to be MORE skeptical and careful before reporting the results, not "run to the press" to publicize these exciting results to get attention to our institutation and the journal. The more dramatic the result, the more cautious we need to be to ensure reproducibility before reporting it! (Cold Fusion :) |
| reproducibility is not an easy concept. Most meaningful things cannot be reproduced. they are unique. every cancer is unique. everything may reproduce in a cell line, but it may not be meaningful. |
| Since many molecular assays haven't been validated - and vagueness in methods is what leads to difficult in reproducibility |
| The answer that I would have given for Question 5 is missing: (1) neither of those journals guarantees the skills that I require; (2) both would be respectable accomplishments for a postdoc applicant, and would be weighted along with many other factors such as recommendations, performance during interview and seminar, etc. I selected 'not faculty' because any other choice would have been too miselading. |
| The Cancer Cell vs Can Res. The most important factor is the quality of the work and its impact in science. Not long ago JBC the Editor wrote about IF. It was obvious that there a number of factors that influence this- so JBC decided not to emphasize review articles and other that have an impact on IF. |
| The impact factor insanity is destroying science. A small group of powerful editors and friends control everything. |
| the previous question about 4 papers in 1 journal vs. 1 in another journal is stupid, it is the quality of the research that counts |
| The previous question is not a good question. It would depend on the resaerch and the candidate. I wouldn't make that decision simply based on number of papers or impact factor of the journals. |
| The survey should bring some rules to influrence or restrict more scientific researchers when they get a nice result but not repeatable, which they should not publish this result. |
| There are numerous biases, many unconscious, that contribute to publication of incorrect data. see Ioannidis JPA (2005) Why Most Published Research Findings Are False. PLoS Med 2(8): e124. doi:10.1371/journal.pmed.0020124 |
| This is a poor survey as you are interested only in cancer research. Your concern should be about all research. |
| This seems like preliminary data. You may like to collect more information on the type of research: basic vs. clinical vs. others, and other variables like size of lab, number of papers published in high , medium and low impact journals; reproducibility of results published; etc. |
| too simplistic a survey to produce meaningful data |
| Very interesting survey. This is a significant problem that is much more widespread than generally accepted. |
| Worked like a charm |
| yes....u didn't ask whether we believe data from our own labs is "reproducible"....or even "real". |
